# Analytical formulas representing track-structure simulations on DNA damage induced by protons and light ions at radiotherapy-relevant energies

**DOI:** 10.1038/s41598-020-72857-z

**Published:** 2020-09-25

**Authors:** Pavel Kundrát, Werner Friedland, Janine Becker, Markus Eidemüller, Andrea Ottolenghi, Giorgio Baiocco

**Affiliations:** 1 Institute of Radiation Medicine, Helmholtz Zentrum München-German Research Center for Environmental Health, Neuherberg, Germany; 2 Department of Radiation Dosimetry, Nuclear Physics Institute CAS, Prague, Czech Republic; 3 Radiation Biophysics and Radiobiology Group, Physics Department, University of Pavia, Pavia, Italy

**Keywords:** Computational biophysics, Biological physics

## Abstract

Track structure based simulations valuably complement experimental research on biological effects of ionizing radiation. They provide information at the highest level of detail on initial DNA damage induced by diverse types of radiation. Simulations with the biophysical Monte Carlo code PARTRAC have been used for testing working hypotheses on radiation action mechanisms, for benchmarking other damage codes and as input for modelling subsequent biological processes. To facilitate such applications and in particular to enable extending the simulations to mixed radiation field conditions, we present analytical formulas that capture PARTRAC simulation results on DNA single- and double-strand breaks and their clusters induced in cells irradiated by ions ranging from hydrogen to neon at energies from 0.5 GeV/u down to their stopping. These functions offer a means by which radiation transport codes at the macroscopic scale could easily be extended to predict biological effects, exploiting a large database of results from micro-/nanoscale simulations, without having to deal with the coupling of spatial scales and running full track-structure calculations.

## Introduction

DNA damage represents the key initial event that underlies biological effects of ionizing radiation^1^. Theoretical approaches valuably complement experimental research on the induction of DNA damage by diverse radiation types under various conditions. In particular, models and simulations help merge information gained from diverse experiments, test putative hypotheses on the action mechanisms, and reveal quantitative characteristics of the underpinning processes^2,3^.

Particularly detailed mechanistic modelling of radiation-induced DNA damage is provided by Monte Carlo track structure simulations (MCTS)^4^. MCTS elucidate the way in which the resulting damage patterns reflect the underlying spatial organization of DNA molecule and chromatin in cell nuclei as well as the hugely varying track structures for diverse types of ionizing radiation. MCTS start from cross-section databases, which capture the probabilities and outcomes of radiation interactions with individual atoms and molecules of the traversed biological medium, typically approximated by liquid water. The cross-section data is sampled using Monte Carlo methods to represent the stochastic nature of radiation interaction with matter. Radiation tracks are simulated, in a given volume of interest, by following all interactions of the primary particle as well as of all the related particles such as secondary and higher-order electrons liberated from the medium. The resulting track structure is then overlaid with a model of cellular DNA and chromatin structures. Energy depositions occurring within this target model are converted to DNA lesions and scored as direct DNA damage. In addition, energy depositions outside the DNA target are considered that produce reactive species via water radiolysis. Diffusion and chemical reactions of these species are followed, especially their attacks on the DNA molecule that lead to its indirect damage.

PARTRAC belongs to the most advanced track-structure tools^5–12^. It uses established cross-section databases for photons, electrons, protons and ions over wide energy ranges relevant for medical, biological and technical applications. It includes a dedicated module for simulating water radiolysis, diffusion and reactions of chemical species. Multi-scale target models are implemented that range from DNA double-helix in atomic resolution to chromosome territories in cell nucleus. Direct and indirect damage is scored and analysed. A subsequent module describes DNA damage repair via non-homologous end-joining of DNA double-strand breaks (DSB), and accounts for the formation of chromosome aberrations. Ongoing work aims at extending the tool towards cell killing. Traditionally, this endpoint of utmost relevance for radiation biology and radiotherapy has been modelled by rather phenomenological approaches that largely avoid addressing the difficult question of what initial events are eventually lethal for the cell^13–19^. Promising preliminary results have been obtained with PARTRAC upon identifying lethal lesions with a subset of chromosomal aberrations (unpublished). PARTRAC simulations have also provided useful information on the initial events that trigger intra- and intercellular communication including bystander effects^20–22^, endpoints which are typically addressed by considerably less detailed models too^23–27^. PARTRAC results on initial radiation-induced DNA damage have been frequently applied as the gold standard for benchmarking other track-structure codes^28–31^. PARTRAC results have also served as input data for the fast Monte Carlo damage simulation tool that enables one to quickly generate damage structures less precisely but considerably faster than full MCTS simulations^32^. Recently, to provide a mechanistic multi-scale model for the biological effectiveness of neutrons, PARTRAC results on DNA damage induced by diverse ions in dependence on their linear energy transfer (LET) have been combined with neutron transport modelled by the macroscopic code PHITS^33,34^. The same approach has been used to help interpret radiobiological measurements of γ-H2AX foci^35^.

To further facilitate the use of PARTRAC results in these rapidly developing areas as well as to provide the necessary input for newly developed repair codes before the recently proposed standard DNA damage format^36^ will be implemented into the tool, in this work we present analytical formulas that capture the results of comprehensive simulations on the initial DNA damage. The formulas reproduce the simulation results on the yields of DNA strand breakage (SB), single-strand breaks (SSB), double-strand breaks (DSB), DSB clusters, and DSB sites upon irradiation with a wide range of ion species and energies relevant for medical applications, from hydrogen to neon and from 512 MeV/u down to their stopping. Since some of the MCTS codes are limited to direct radiation effects only, for benchmarking purposes we report in addition to the total yields also damage yields from direct and indirect effects separately.

## Methods

The previously published database of PARTRAC results on initial DNA damage by light ions^11^ was extended to 512 MeV/u and complemented by Li, Be and B ions; the first results published in^12^ were complemented here by additional calculation runs for protons at the highest energies to improve the statistics for rather rare DSB clusters. The simulation results were fitted by appropriate analytical functions. The most pertinent methodological issues are described below; further details on the methods used in PARTRAC can be found in^2,6,11,12,37^ and references therein.

### Irradiation setup

A spherical model of human lymphocyte nuclei (10 µm diameter) containing 6.6 Gbp DNA in 23 chromosome pairs, corresponding to the interphase (G0/G1) cell cycle phase, was in-silico irradiated by ^1^H, ^4^He, ^7^Li, ^9^Be, ^11^B, ^12^C, ^14^N, ^16^O or ^20^Ne ions. The ions were started from random locations within a circular source (10.09 µm diameter) tangential to the cell nucleus, with directions perpendicular to the source plane. Note that this setup does not provide electronic equilibrium conditions (cf. “Discussion”). To avoid alignment with direction axes of the chromatin model, the source was randomly rotated in 3D with respect to the nucleus structure. The ions were fully charged, i.e. completely stripped of electrons, with initial energies of 512, 256, 128, 64, 32, 16, 8, 4, 2, 1, 0.5 or 0.25 MeV/u. To account for secondary electrons leaving and possibly re-entering the nucleus, interactions were scored in a spherical region with a diameter of 14.22 µm concentric with the nucleus model. For each ion species and starting energy, at least 1280 particles were simulated (up to 3.2 million in the case of high-energy protons), divided into 256–8192 simulation runs with 5–3200 particles started in each run.

### Interaction cross sections

Established cross sections were used for electromagnetic interactions of H and He in liquid water that explicitly account for charge-changing processes, relevant especially for energies < 1 MeV/u, and distinguish two states for hydrogen (neutral hydrogen atoms H^0^ and protons H^+^) and three charge states for helium (He^0^, He^+^, He^2+^). For ions heavier than He, electronic cross sections were scaled from those of H (energy-dependent H^0^/H^+^ mixture) at the same energy per unit mass using the ion’s energy-dependent effective charge^11,37^; for energies > 1 MeV/u, this reproduced the standard Barkas scaling of proton cross sections. Nuclear reactions as well as lateral scattering of ions in electronic interactions were neglected, as done standardly in PARTRAC; thus, ions were assumed to travel along straight lines. Interactions of all secondary and higher-order electrons liberated by the primary particles or by lower-order electrons were followed until leaving the region of interest; the implemented cross sections account for five ionization and five excitation levels of liquid water as well as for elastic scattering.

### Dose and linear energy transfer (LET)

Yields of DNA damage are typically expressed per unit dose (Gy) and per gigabasepair (Gbp) of cellular DNA. Dose applied to the nucleus was evaluated by summing up energy depositions from the primary ion as well as all secondary and higher-order electrons within the spherical cell nucleus, and by dividing them by its mass. For low-energy ions whose ranges are smaller than the diameter of the cell nucleus, only the respective irradiated spherical caps were considered. The deposited doses per run ranged from 20 mGy to 15 Gy; the total dose deposited in all simulations for a given ion and its starting energy ranged from 72 Gy to 5.4 kGy (on average 920 Gy).

To characterize radiation quality with LET, the scored dose was divided by particle fluence^11^. As discussed in detail there, this LET may be used to characterize the given radiation type over the scale of the nucleus (or its irradiated part, respectively).

### DNA damage

Standard PARTRAC assumptions on the biophysics and biochemistry of the DNA molecule were used that govern its direct and indirect breakage^6,11^: the probability to induce a strand break (SB) linearly increased from 0 at 5 eV to 1 at 37.5 eV deposited to a single sugar-phosphate group (direct effects), and amounted to 65% for ^•^OH attacks on the deoxyribose (indirect effects), while no other species were assumed to induce SB. Standard damage classification scheme was used: SB on both strands within 10 bp were scored as a DSB, two or more DSB within 25 bp as a DSB cluster, and both an isolated DSB and a cluster were scored as a single DSB site^11^. SB being not part of DSB were scored as single-strand breaks (SSB). Base damage was not considered. The lesions were scored and analysed in a track-by-track manner, i.e. neglecting potential inter-track effects (e.g., SB from two distinct primary particles combining into a DSB), as done standardly in PARTRAC. This approach corresponds to the basic concept of reporting the damage yields per unit dose. The linearity of damage induction with applied dose holds unless independent tracks overlap both spatially (on nm scales) and temporally (on ns scales), i.e. at least until doses of the order of 100–1000 Gy^38^, well above the simulated dose per run.

### Analytical fits of PARTRAC results

For the sake of consistence with radiobiological studies, the dependence of damage yields on LET was analysed, rather than that on the starting energy of the ion. LET has also served as the interface between macroscopic transport calculations with PHITS and micro-/nanoscopic DNA damage simulations with PARTRAC in previous studies^33,34^. The drawback of this LET-based approach is that LET-dependent damage yields show hooks that cannot be represented by a single function (cf. “Results”): LET is not a monotonous function of the ion starting energy but possesses a maximum (at ion energy of about 1 MeV/u). A given LET value can be found in both proximal and distal parts of the Bragg peak. At the same LET, the lower-energy ion liberates slower electrons and hence possesses a narrower but denser track than the higher-energy ion. Hence their effects including the induction of DNA damage are different, which manifests as hooks in LET-dependent DNA damage presented below. Therefore, the corresponding simulation results at the lowest energies were not considered in the analytical fits (0.25 MeV/u for H, He, Li, Be and B ions, and 0.5 and 0.25 MeV/u for C, N, O and Ne ions). According to the trends observed in the analysed results of PARTRAC simulations, the following appropriate model functions were chosen:

The simulated LET-dependent yields of SB and SSB were fitted by

1 $${\text{Yield}} = {\text{p}}_{1} {-}\left( {{\text{p}}_{2} {\text{LET}}} \right)^{{{\text{p}}_{3} }} {-}{{{\text{p}}_{4} } \mathord{\left/ {\vphantom {{{\text{p}}_{4} } {\left( {1 + \log^{2} \left( {{\text{LET/p}}_{5} } \right)} \right)}}} \right. \kern-\nulldelimiterspace} {\left( {1 + \log^{2} \left( {{\text{LET/p}}_{5} } \right)} \right)}}.$$

where Yield is in Gy^−1^ GBp^−1^ and LET in keV μm^−1^. This formula works with five parameters: p_1_ depicts the low-LET damage yield, which is followed by a power-law decrease with increasing LET (parameters p_2_, p_3_), and the last term (parameters p_4_, p_5_; log denotes natural logarithm) accounts for a slight dip in SB and SSB yields at LET in the intermediate range of approximately 5–20 keV/µm predicted by the simulations.

The simulated LET-dependent yields of DSB, DSB clusters and DSB sites were fitted by

2 $${\text{Yield}} = ({\text{p}}_{1} + {{\left( {{\text{p}}_{2} {\text{LET}}}\right)^{{{\text{p}}_{3} }} }) \mathord{\left/ {\vphantom {{\left( {{\text{p}}_{2} {\text{LET}}} \right)^{{{\text{p}}_{3} }} } {\left( {1 + \left( {{\text{p}}_{4} {\text{LET}}} \right)^{{{\text{p}}_{5} }} } \right)}}} \right. \kern-\nulldelimiterspace} {\left( {1 + \left( {{\text{p}}_{4} {\text{LET}}} \right)^{{{\text{p}}_{5} }} } \right)}}.$$

where Yield is in Gy^−1^ GBp^−1^ and LET in keV μm^−1^. Also this function uses five adjustable parameters: p_1_ captures the low-LET damage yield, which is followed by a power-law increase with increasing LET (parameters p_2_, p_3_), modulated by a logistic decrease that reflects the so-called ‘overkill’ effect at high LET (parameters p_4_, p_5_).

These functions were used separately for each damage class considered, and separately for the yields from purely direct and purely indirect effects, as well as for the total yields. This was necessary since the total damage yields could not be modelled by the sum of the direct and indirect contributions: occasionally an indirectly induced SB (upon an ^•^OH attack) would occur at the location where a direct SB (upon sufficient energy deposition) has already happened. While such events are tallied to both direct and indirect SB when these are scored separately, they contribute to the total SB yield as a single event only. In addition, for DSB classes, ‘hybrid’ events occur quite frequently, where a direct SB on one strand combines with an indirect SB on the other strand.

The low-LET yields (parameters p_1_ in both formulas) were adjusted manually, separately for each of the modelled DNA damage classes but universally for all the studied ions. The remaining parameters were fitted for each damage class and each ion separately, using nonlinear regression tools in Matlab (The MathWorks Inc., USA). For some ion species and damage classes, the fit formulas were simplified by omitting a term for which there was no indication in the results of PARTRAC simulations; this was the case e.g. for proton-induced DSB classes for which no ‘overkill’ was predicted by the simulations.

## Results

### DNA strand breakage

**Figure 1:**
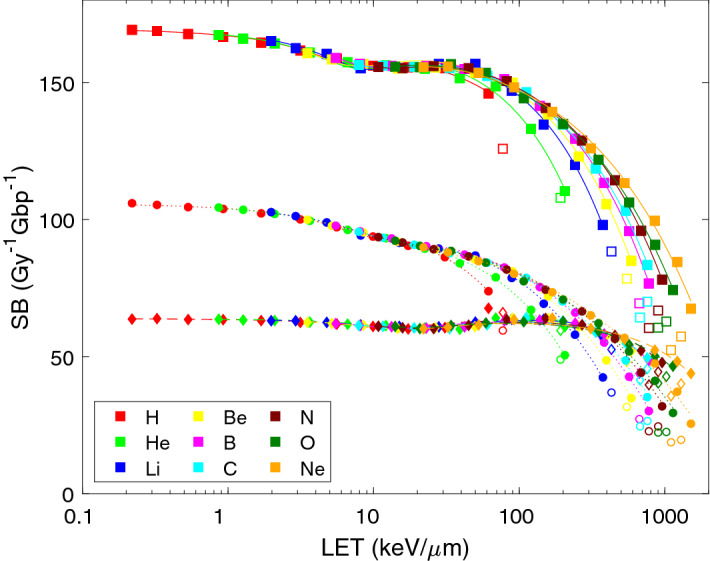
PARTRAC simulations of LET-dependent strand breakage (SB) upon irradiation with light ions. Symbols show the results of track-structure simulations, lines their analytical representation using formula (1) with fit parameters listed in Table 1. Presented is SB induced via direct effects (diamonds, dashed lines), indirect effects (circles, dotted lines) and total yields (squares, solid lines). Simulation results for the lowest energies (empty symbols) were not included in the analytical fits.

The results of PARTRAC simulations on DNA strand breakage (SB) and their analytical representations are presented in Fig. 1. Direct energy depositions within the DNA molecule (direct effects; simulation results depicted by diamonds, analytical fits by dashed lines) are predicted to induce about 64 SB per Gy per Gbp for low-LET (high-energy) ions. Actually, the direct SB yield is largely independent of ion type and energy except for high-LET (low-energy) particles, for which the yield decreases in an ion-specific manner, with lowest figures around 35–40 directly induced SB per Gy per Gbp. While direct SB yields are largely constant up to 100 keV/µm, there is a slight but notable dip at intermediate LET values around 20 keV/µm. The yields of indirect SB (circles and dotted lines), resulting from ^•^OH attacks on the DNA, are almost twice higher (about 106 SB per Gy per Gbp) than direct ones for low-LET (high-energy) particles. With increasing LET, however, the indirect SB yields gradually decrease due to increasing recombination of reactive species within particle tracks as the tracks become narrower and denser (track thin-down due to the reduced maximum energy and hence range of secondary electrons); at low energies (high LET), this decrease is ion-specific. In addition to this general trend described by a power-law decrease, there is a mild dip in indirect SB numbers at LET around 10 keV/µm, largely independent of ion type. At the lowest energies, direct strand breakage dominates over the indirect one; the latter drops down to e.g. 19 indirect SB per Gy per Gbp for 0.25 MeV/u neon ions. Also the total SB yields (squares and solid lines) in general show a power-law decrease with increasing LET, with a mild dip at around 10 keV/µm. Also the total SB yields are largely ion type-independent at high energies but ion-specific at low energies (high LET). Model parameters of the analytical representations [formula (1)] of the simulation results are listed in Table 1 (upper part), for total yields as well as direct and indirect effects separately. The yields of total SB decrease proportionally to LET to the powers (p_3_) of 0.5–0.9, i.e. roughly proportionally to the square root of LET to linearly with LET, apart from the mild dips. This holds for indirect SB too. Direct SB are largely constant up to LET > 100 keV/µm where they, except for H and He, drop in a supra-linear manner (p_3_ = 1.1–3.4).

**Table 1:** Parameters of formula (1) representing track-structure simulations on DNA strand breakage (SB) and induction of DNA single-strand breaks (SSB) by H to Ne ions.

Damage class	Ion	Total yields					Direct effects					Indirect effects				
		p_1_	p_2_	p_3_	p_4_	p_5_	p_1_	p_2_	p_3_	p_4_	p_5_	p_1_	p_2_	p_3_	p_4_	p_5_
SB	H	170	1.335	0.7023	8.541	6.902	64	N.A.	N.A.	3.532	12.51	106	1.076	0.8189	5.679	9.223
	He	170	0.4632	0.8913	11.81	7.542	64	N.A.	N.A.	4.015	20.46	106	1.815	0.6758	5.652	13.93
	Li	170	0.405	0.8499	12.25	8.795	64	0.004687	3.354	3.525	15.07	106	1.784	0.6373	6.574	13.43
	Be	170	0.6563	0.7454	10.84	7.203	64	0.005799	2.023	4.085	16.17	106	3.298	0.5625	5.735	13.88
	B	170	0.7101	0.7173	10.36	9.02	64	0.006684	1.662	3.704	15.59	106	4.198	0.5332	4.87	13.1
	C	170	0.9285	0.6785	10.02	9.499	64	0.006881	1.621	3.802	16.69	106	6.272	0.5007	4.717	15.26
	N	170	0.9985	0.6579	9.627	11.12	64	0.006951	1.485	3.747	18.77	106	7.44	0.4832	4.843	17.03
	O	170	1.754	0.5993	9.64	8.154	64	0.01046	1.168	3.224	19.08	106	14.64	0.4437	3.151	15.89
	Ne	170	2.388	0.5616	8.841	9.224	64	0.009527	1.127	3.748	18.59	106	20.28	0.4207	3.219	28.1
SSB	H	156	0.9613	0.9173	10.21	7.124	60	39.79	0.2471	4.189	35.17	102	2.438	0.7084	4.389	7.916
	He	156	1.681	0.7616	9.093	8.052	60	1.765	0.5268	1.127	1.642	102	3.242	0.6263	4.999	12.97
	Li	156	1.856	0.7023	9.737	8.993	60	0.3056	0.7	4.357	8.53	102	3.067	0.5961	6.479	13.54
	Be	156	3.771	0.6105	7.937	7.851	60	0.2586	0.6959	5.034	11.37	102	6.137	0.5251	5.421	14.58
	B	156	5.414	0.5695	6.837	8.997	60	0.298	0.6564	4.575	12.74	102	9.016	0.4909	4.265	14.22
	C	156	9.511	0.5256	5.818	8.409	60	0.3865	0.6131	4.429	16.03	102	14.25	0.4602	4.069	16.06
	N	156	12.73	0.5006	5.67	8.453	60	0.3666	0.605	4.612	19.38	102	19	0.4397	4.008	17.7
	O	156	21.17	0.4693	4.926	6.674	60	0.651	0.5414	3.499	20.59	102	40.6	0.4036	2.215	15.94
	Ne	156	52.47	0.4215	N.A.	N.A.	60	0.8246	0.5072	3.615	24.24	102	65.9	0.3788	2.26	30.59

Parameter p_1_ (low-LET yields) was adjusted universally at the same value for all ions, the other 4 parameters (p_2_, …, p_5_) were fitted for each ion species separately. In addition to total damage yields, parameters describing direct and indirect effects are provided separately. N.A., not applicable: corresponding term not indicated by the simulation results and hence not included in the given case.

**Figure 2:**
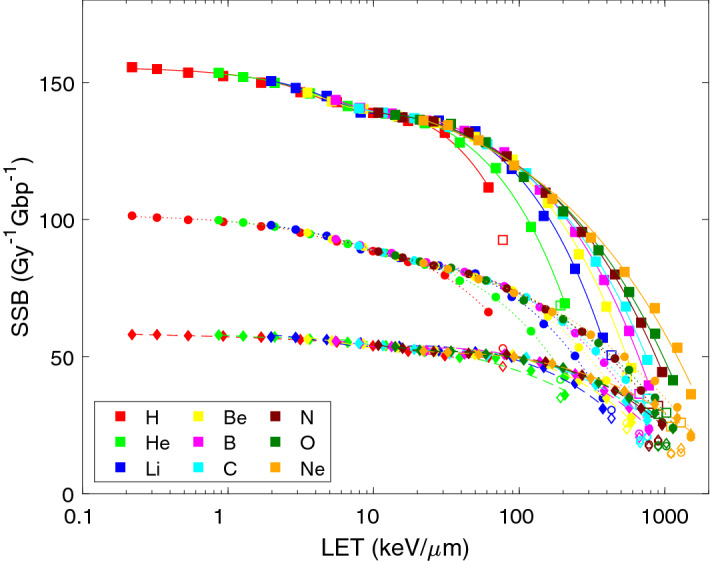
Results of PARTRAC simulations on LET-dependent yields of single-strand breaks (SSB) upon irradiation with light ions. Symbols show the results of track-structure simulations, lines their analytical representation using formula (1) with fit parameters listed in Table 1. Presented are SSB induced via direct effects (diamonds, dashed lines), indirect effects (circles, dotted lines) and total yields (squares, solid lines). Simulation results for the lowest energies (empty symbols) were not included in the analytical fits.

Most of the induced strand breaks are isolated as single-strand breaks (SSB), over the whole studied range of ion species and starting energies except for 0.25 or 0.5 MeV/u C, N, O or Ne ions. SSB yields are presented in Fig. 2. Again, indirect effects (circles and dotted lines) contribute to the total SSB induction (squares and solid lines) almost twice more than direct effects (diamonds and dashed lines) for high-energy (low-LET) ions. While direct effects remain largely constant with decreasing ion energy (increasing LET), indirect SSB induction drops down, and finally direct effects dominate for low-energy (high-LET) ions. Similarly to SB, the functional dependences of direct, indirect as well as total SSB on LET obey power-law decreases with increasing LET, modulated by mild dips at LET values around 5–20 keV/µm (somewhat shallower than in the case of SB). The corresponding parameters of formula (1) are listed in Table 1 (bottom part). Similarly to SB, apart from the mild dips, the yields of SSB from direct and indirect effects combined as well as only from indirect effects decrease roughly with the square root to linearly with LET (p_3_ = 0.4–0.9), while SSB yields from direct effects are almost constant up to rather high LET values (~ 100 keV/µm) but then decrease sub-linearly with LET (p_3_ = 0.2–0.7).

### Double-strand breaks and their clustering

**Figure 3:**
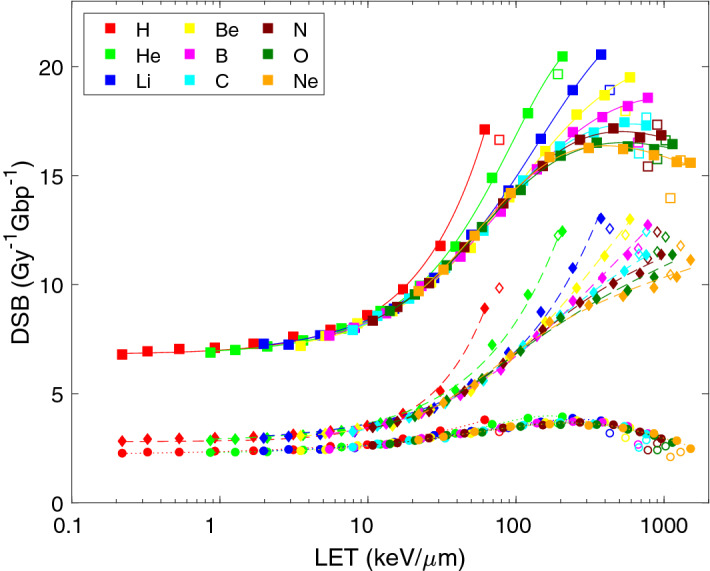
LET-dependent yields of double-strand breaks (DSB) upon irradiation with light ions. Symbols show the results of track-structure simulations, lines their analytical representation using Eq. (2) with fit parameters listed in Table 2. Presented are DSB induced via direct effects (diamonds, dashed lines), indirect effects (circles, dotted lines) and total yields (squares, solid lines). Simulation results for the lowest energies (empty symbols) were not included in the analytical fits.

A well-known hallmark of ionizing radiation is the stochastic but clustered energy deposition. As a consequence, not all strand breaks are isolated as SSB, but also DNA double-strand breaks (DSB) occur that are formed by strand breaks on both strands of the DNA molecule in a close vicinity (10 bp in this study). As shown in Fig. 3, low-LET ions are predicted to induce about 7 DSB per Gy per Gbp. About 40% of this yield is due to direct effects (both strand breaks induced directly; diamonds and dashed lines), in about 30% both strand breaks forming the DSB are due to indirect ones (circles and dotted lines), and the rest corresponds to ‘hybrid’ cases that combine a direct and an indirect strand break. With increasing LET, the total DSB yields (squares and solid lines) increase, and reach values as high as 20 DSB per Gy per Gbp for low-energy light ions. The contribution of direct effects increases with increasing LET. At the same LET, protons and helium ions are more effective in inducing DSB than heavier ions. With increasing atomic number, the differences between ion types tend to diminish, apart from ion-specific hooks at the lowest energies. Parameters of the analytical formula (2) that reflect the DSB-LET relationships predicted by PARTRAC simulations are listed in Table 2 (upper part). Up to the overkill above ~ 100 keV/µm, direct and total DSB yields increase with increasing LET approximately in a linear manner (p_3_ ~ 1), indirect ones in a sub-linear way (p_3_ = 0.6–0.7).

**Table 2:** Parameters of formula (2) representing track-structure simulations on the induction of DNA double-strand breaks (DSB), their clusters and DSB sites by H to Ne ions.

Damage class	Ion	Total yields					Direct effects					Indirect effects				
		p_1_	p_2_	p_3_	p_4_	p_5_	p_1_	p_2_	p_3_	p_4_	p_5_	p_1_	p_2_	p_3_	p_4_	p_5_
DSB	H	6.8	0.1835	0.9583	N.A.	N.A.	2.8	0.07011	1.231	N.A.	N.A.	2.2	0.03598	0.5834	N.A.	N.A.
	He	6.8	0.1679	0.9704	0.004323	1.359	2.8	0.08076	0.816	N.A.	N.A.	2.2	0.02683	0.6349	0.002725	2.022
	Li	6.8	0.2148	0.864	0.00399	0.9872	2.8	0.07501	0.7078	N.A.	N.A.	2.2	0.03443	0.6439	0.002556	1.057
	Be	6.8	0.2148	0.9999	0.009586	1.019	2.8	0.08651	0.9131	0.003924	0.8367	2.2	0.03583	0.7321	0.003678	1.088
	B	6.8	0.2303	0.9711	0.009576	1.016	2.8	0.1168	0.9562	0.009723	0.8111	2.2	0.03316	0.7289	0.003393	1.133
	C	6.8	0.2052	1.02	0.009922	1.106	2.8	0.09374	1.076	0.01033	1.006	2.2	0.03152	0.6538	0.002669	1.114
	N	6.8	0.2043	1.023	0.01002	1.121	2.8	0.09184	1.103	0.01089	1.05	2.2	0.03022	0.6392	0.002471	1.13
	O	6.8	0.2122	1.077	0.01311	1.146	2.8	0.108	1.184	0.01773	1.088	2.2	0.03304	0.6638	0.002959	1.085
	Ne	6.8	0.1916	1.112	0.01261	1.204	2.8	0.09018	1.34	0.01838	1.263	2.2	0.0308	0.5917	0.002172	1.081
DSB clusters	H	0.07	0.01532	2.396	N.A.	N.A.	0.018	0.01152	2.844	N.A.	N.A.	0.004	0.002534	1.952	N.A.	N.A.
	He	0.07	0.01015	1.794	0.003817	3.255	0.018	0.006072	1.762	N.A.	N.A.	0.004	0.00101	1.464	N.A.	N.A.
	Li	0.07	0.008907	2.004	0.004511	2.064	0.018	0.005925	2.183	0.004068	1.607	0.004	0.0006276	1.35	N.A.	N.A.
	Be	0.07	0.007692	1.736	0.003448	2.088	0.018	0.00427	1.746	0.002058	2.09	0.004	0.00048	1.242	0.001365	5.796
	B	0.07	0.007604	1.726	0.003789	1.991	0.018	0.004394	1.817	0.002721	1.85	0.004	0.0003195	1.1	0.001242	37.32
	C	0.07	0.006858	1.498	0.002778	2.208	0.018	0.003932	1.678	0.002187	2.14	0.004	0.0005724	1.265	0.001473	1.094
	N	0.07	0.00661	1.418	0.002577	2.194	0.018	0.003585	1.519	0.00184	2.216	0.004	0.0004915	1.207	0.00144	0.9436
	O	0.07	0.007119	1.514	0.003193	2.095	0.018	0.003711	1.533	0.002088	2.046	0.004	0.0005992	1.322	0.002002	1.108
	Ne	0.07	0.006894	1.418	0.002865	2.108	0.018	0.003522	1.485	0.001971	2.097	0.004	0.001511	1.71	0.007381	1.29
DSB sites	H	6.8	0.1773	0.9314	N.A.	N.A.	2.8	0.06901	1.196	N.A.	N.A.	2.2	0.035	0.5841	N.A.	N.A.
	He	6.8	0.1471	1.038	0.006239	1.582	2.8	0.06555	1.023	0.003748	1.763	2.2	0.02656	0.6415	0.002875	1.994
	Li	6.8	0.1653	0.8782	0.004284	1.406	2.8	0.06093	0.9556	0.003178	1.402	2.2	0.03349	0.6485	0.002736	1.109
	Be	6.8	0.1425	0.95	0.005151	1.407	2.8	0.06199	0.9224	0.003301	1.322	2.2	0.0341	0.7328	0.003678	1.136
	B	6.8	0.1587	0.8714	0.004345	1.389	2.8	0.063	0.9255	0.003655	1.305	2.2	0.03117	0.7196	0.003265	1.182
	C	6.8	0.156	0.9214	0.005245	1.395	2.8	0.06191	0.9903	0.004525	1.369	2.2	0.02946	0.6435	0.002585	1.166
	N	6.8	0.1641	0.875	0.004607	1.391	2.8	0.06171	0.9649	0.004156	1.389	2.2	0.02776	0.6216	0.00231	1.189
	O	6.8	0.1749	0.8722	0.004987	1.347	2.8	0.0664	0.969	0.004754	1.341	2.2	0.03004	0.6396	0.002652	1.136
	Ne	6.8	0.1797	0.8657	0.004917	1.346	2.8	0.06408	1.023	0.0053	1.386	2.2	0.02847	0.5684	0.002006	1.128

Parameter p_1_ (low-LET yields) was adjusted universally at the same value for all ions, the other 4 parameters (p_2_, …, p_5_) were fitted for each ion species separately. In addition to total damage yields, parameters describing direct and indirect effects are provided separately. N.A., not applicable: corresponding term not indicated by the simulation results and hence not included in the given case.

**Figure 4:**
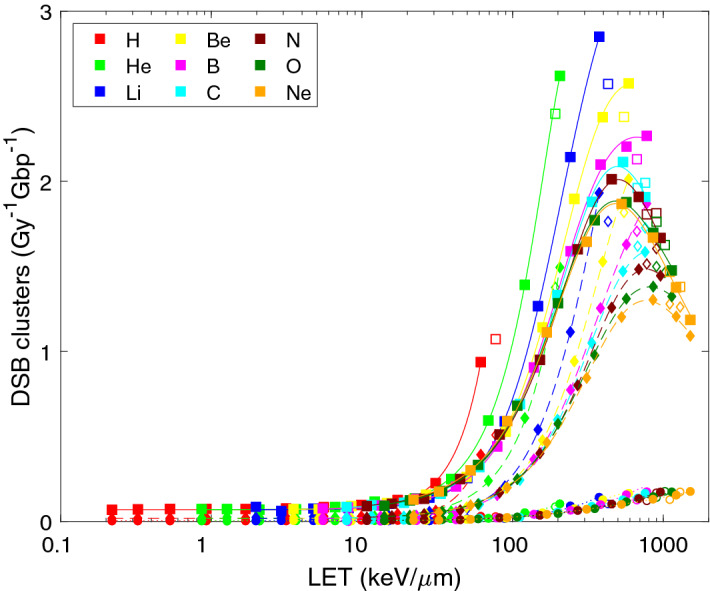
LET-dependent yields of clusters of double-strand breaks (DSB clusters) upon irradiation with light ions. Symbols show the results of track-structure simulations, lines their analytical representation using Eq. (2) with fit parameters listed in Table 2. Presented are DSB induced via direct effects (diamonds, dashed lines), indirect effects (circles, dotted lines) and total yields (squares, solid lines). Simulation results for the lowest energies (empty symbols) were not included in the analytical fits.

Also DSB tend to cluster. Using the definition of DSB cluster as at least two DSB separated by less than 25 bp, the simulated yields of DSB clusters are shown in Fig. 4. While cluster yields at low LET of approximately 0.07 per Gy per Gbp correspond to only about 1% of DSB, with increasing LET the induction of DSB clusters rapidly increases, and finally more than 10% of DSB form clusters. At highest LET values of carbon and heavier ions, there are on average more than five DSB per cluster^11^. Parameters of formula (2) adjusted to the simulated DSB cluster data are summarized in Table 2 (middle part). The yields of DSB clusters from indirect, direct and total effects all increase with LET in a supra-linear manner (sub-quadratic to quadratic, p_3_ ranging from 1.1 to 2.8, for protons even supra-quadratic, p_3_ from 2 to 2.8).

**Figure 5:**
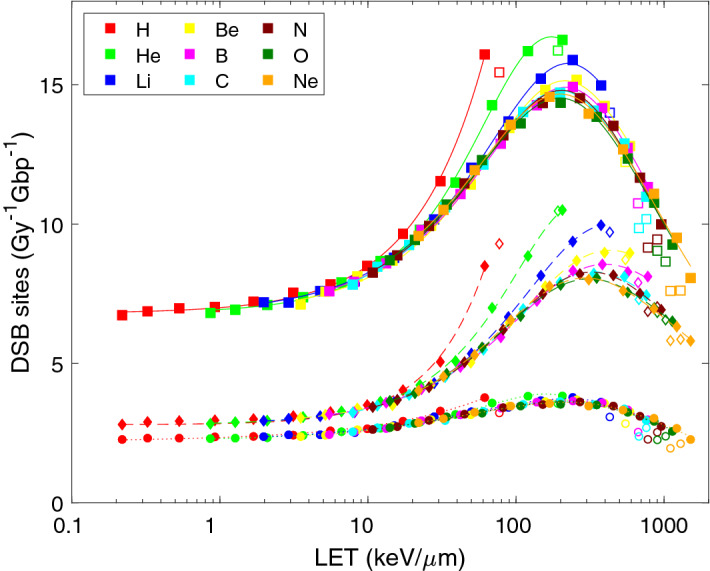
LET-dependent yields of double-strand break sites (DSB sites) upon irradiation with light ions. Symbols show the results of track-structure simulations, lines their analytical representation using Eq. (2) with fit parameters listed in Table 2. Presented are DSB induced via direct effects (diamonds, dashed lines), indirect effects (circles, dotted lines) and total yields (squares, solid lines). Simulation results for the lowest energies (empty symbols) were not included in the analytical fits.

When sites of isolated DSBs or their clusters are scored, their LET dependence (Fig. 5) closely resembles the well-known pattern observed in cell survival data, reviewed in^39^. Again, protons and helium ions are more effective in inducing DSB sites than heavier ions at the same LET. Data for heavier ions largely follow a universal, ion-independent pattern. The effectiveness in the induction of DSB sites peaks at about 15 sites per Gy per Gbp for LET values of 100–200 keV/μm, which about doubles the yields by low-LET particles. Also in this case the simulation results can be reproduced by formula (2), with parameters listed in Table 2 (bottom part). The yields of DSB sites from direct and total effects increase with LET about linearly (p_3_ ~ 1), from indirect effects sub-linearly (p_3_ = 0.6–0.7).

### Uncertainty and fit quality

Statistical uncertainties on the total yields of the mentioned DNA damage classes except DSB clusters were quite low, with coefficients of variation within 1.5% (direct and indirect yields separately within 2.7%). For DSB clusters, which are rather rare events especially for high-energy ions, the uncertainty of total yields was within 10% (on average 3.7%), except for Li ions > 10 MeV/u where it amounted to 10–15%.

The fits reproduced the simulations with a very high accuracy, with root-mean-square relative deviation within 2% for each ion and each damage class except DSB clusters (up to 9%).

## Discussion

The reported analytical formulas accurately reproduce the results of full PARTRAC MCTS simulations on the initial DNA damage induction by ions from hydrogen to neon at energies from 512 down to 0.5–1 MeV/u, in dependence on the LET as used commonly in radiation biology. The present results show that the yields of DNA damage largely obey a power-law dependence on LET, with ion type-dependent power. The yields of SB and SSB decrease proportionally to LET to the powers of 0.5–1 (i.e. proportionally to the square root of LET to linearly with LET), apart from mild dips at LET values around 10–20 keV/µm. The total yields of DSB and DSB sites increase about linearly with LET (the values of p_3_ are close to unity, Table 2). For DSB clusters, this increase with LET is sub-quadratic to quadratic (p_3_ ranging from 1.4 to 2.4, Table 2), for protons even supra-quadratic (p_3_ = 2.4). For all DSB classes, ion-specific overkill occurs for LET above 100 keV/µm.

These trends arise from the complex interplay of energy deposition patterns by the primary particle and all its secondary electrons, water radiolysis, radical diffusion and their mutual reactions, and the overlap of these structures with local DNA organisation, all of which are accounted for by PARTRAC MCTS simulations. LET describes how much energy is deposited per unit track length; neither the lateral track profile nor the stochastic track structure are reflected by this quantity. Yet, despite its simplicity, it is a useful descriptor of radiation quality with respect to biological effects. This is perhaps best illustrated by the linear nature of the LET-effect relationship for DSB and DSB sites and the link of these damage classes to cell killing.

The amount of energy deposited per track is proportional to its LET. Also the probability to deposit sufficient energy to the sugar-phosphate moiety of the DNA molecule and thus to induce a direct strand breakage increases with LET. The present results show that the ratio of these two endpoints, i.e. direct strand breakage per unit dose, is largely constant, independent of LET. This may likely be traced back to the key contribution of electron track ends for all ions. This independence of strand breakage on LET holds up to very high LET values (~ 200 keV/µm). There, additional energy deposits to the same group may occur without further enhancing the breakage effectiveness, an effect that could be called ‘over-breakage’. Strand breakage by ^•^OH attacks decreases with increasing LET, predominantly due to increasing recombination of radicals within denser tracks. Although radical attacks on sugar-phosphate groups already broken by direct effects are scored as indirect strand breakage as well, the total strand breakage can be approximated by the sum of direct plus indirect effects, which explains the reported dependence on LET. SSB yields follow largely similar trends as those of strand breakage, but decrease with LET somewhat faster as SB clustering into DSB increases with increasing LET. For both SB and SSB, minor dips at LET values around 10–20 keV/µm are predicted by the PARTRAC MCTS simulations. These dips amount to 3–7% of the yields. Further investigation is needed to clarify whether they represent a mere artefact of the simulations or might be related e.g. to the lack of electronic equilibrium conditions at high starting energies of the ions (i.e., relatively low LET values) in the given setup.

The approximately linear increase in DSB yields with LET can be understood similarly to the independence of SB yields on LET: The total strand breakage (not per unit dose) increases about proportionally to LET. DSB combine pairs of SB, and thus their absolute yields increase about quadratically with LET. As also the deposited dose increases linearly with LET, the DSB yield per unit dose is approximately proportional to LET. This holds roughly also for DSB sites, since isolated DSB dominate over their clusters. As DSB clusters combine at least two DSB, they increase with LET in a supra-linear manner.

The above discussion summarizes simplified arguments to explain the major trends in radiation-induced DNA damage induction. Similar but considerably more detailed considerations represent the basis of other modelling or theoretical approaches, e.g. the multi-scale approach (MSA) reviewed in^40^. The detailed MCTS simulations reported in this work and the trends reflected by the analytical formulas may provide a useful benchmark for such theoretical approaches and their underlying assumptions and approximations.

While well suited for interpolation purposes, the reported analytical LET-dependent formulas should not be extrapolated outside the shown ranges. In particular, the formulas do not account for hooks at energies below 1 MeV/u, which appear due to ions on proximal and distal parts of the energy-loss curve possessing the same LET but distinct radial track characteristics and hence effectiveness. While the differences are minimal in some cases (e.g. for 0.25 MeV He ions), in other cases the analytical formulas deviate from MCTS results by more than 10% (e.g. for 0.25 MeV Ne ions). Such data could be represented if the particles were characterized by their energy rather than LET, since no hooks appear in such representation^11^. As already mentioned, LET has been used as the descriptor of radiation quality here for the sake of consistence with its common use in radiobiology. However, for linking micro- and nanoscale MCTS simulations of biological effects with macroscopic transport codes and for enabling multi-scale simulations down to particle stopping, model description in terms of particle energy and atomic number might be more suitable.

The given choice of functional forms was motivated by the trends observed in the PARTRAC MCTS simulation results and by the sake of simplicity. Power-law expressions were combined with a logistic formula to account for the reduced effectiveness (‘overkill effect’) in the induction of DSB classes at high LET, or modulated by a Gaussian bell-shaped function in the logarithmic LET scale to represent the small dip in SB and SSB at LET values of 5–20 keV/µm predicted by the simulations. Obviously, alternative fit functions such as exponentials^33^ or polynomials in log–log scale might have been used as well, but were found inferior in terms of their ability to reproduce the simulation data (results not shown). While the low-LET yields were considered as species-independent parameters (and adjusted manually), other parameters were fitted for each ion species specifically. The general trends in the power-law and overkill parameters indicate that at least a phenomenological model if not a full theory could likely be formulated that would include the dependence on ion charge as well. Such a model would describe the initial DNA damage with a few universal parameters instead of four ion-specific ones used here.

Developing such a new phenomenological or theoretical approach exceeds far beyond the scope of this work. We recall that the ion-specific analytical formulas presented here are representations of full MCTS results, and as such are sufficient for facilitating the applications of PARTRAC, the aim of this work. A full comparison to other approaches leading to calculations of radiation-induced damage at the subcellular level up to the prediction of cell survival as the relevant outcome for ion-beam cancer therapy applications would also deserve dedicated efforts.

In addition to total yields of diverse DNA damage classes, we have separately reported damage yields from direct and indirect effects and their analytical fits. In particular, this will facilitate using the present results for benchmarking codes that are limited to direct effects only.

The reported analytical formulas may also provide the needed input for alternative repair models before making PARTRAC results available in the recently established standard DNA damage format^36^. The reported simulations and their analytical approximations may also inform analytical models of radiation-induced cell killing^17,18^.

The given simulation setup corresponds to track-segment irradiation without reaching electronic equilibrium, e.g. to irradiation of cells covered by a minimal water layer only. Detailed PARTRAC MCTS simulations with varying thickness of water layer traversed before reaching the cell nucleus, which gradually grant the conditions of electronic equilibrium, are computationally very expensive. Nevertheless, a pilot study has shown that the per-Gy damage yields are very well approximated by the present setup, the deviations being limited to 2% for SSB and 4% for DSB^12^. Thus the present simulation results and their analytical approximations could be used also for modelling radiation effects in macroscopic volumes. Using MCTS in conjunction with macroscopic transport simulations^41,42^ is faster than applying MCTS over large-scale volumes, and enables accounting for nuclear reactions and lateral scattering of ions that are commonly neglected in MCTS. Representing MCTS results by analytical functions further speeds up such models; recently, such an approach has been employed to model the biological effectiveness of neutrons^33–35^.

It is also important to recall that the present results have been based on simulations with a single model of a spherical cell nucleus in G0/G1 cell cycle phase. However, the results are largely insensitive to variations in cell shape or size: DNA damage yields from direct effect are almost completely determined by target size (sugar-phosphate moiety) in relation to the track structure on nm scales, with only a limited impact of DNA compactness (hetero- vs euchromatin) via the different volumes of DNA hydration shell contributing to quasi-direct effects^11^. For indirect effects, this impact is clearly more pronounced. The extent of radical formation and scavenging may largely vary with the degree of chromatin compaction. Unfortunately, to our knowledge experimental data are missing that could be used as a solid basis for corresponding simulations. We have previously reported a marginal but significant difference between lymphocytes and fibroblasts^11^, but this has been based on differences in track structure features within the cellular volume (such as ion stopping and relation between effects of the primary ion and of secondary electrons). Anyway, the size and shape of the nucleus play a more important role for effects at larger scales such as large fragments and chromosome aberrations, not for the local effects (SB, SSB, DSB and their sites and clusters) reported in this work. Cell-cycle dependence is not addressed by PARTRAC at all, since the tool includes G0/G1 chromatin models only.

Though not specifically discussed in this work, the proposed formulas can be used to extract values of relative biological effectiveness (RBE) for the induction of different types of DNA damage. When the specific DNA damage type under consideration shows a linear dependence on radiation dose, as is the case here, the RBE (formally defined as the ratio of doses at the same effect) is simply given by the ratio of damage yields at the same dose. As done previously^11^, the simulation results for low-LET hydrogen ions can be used as a reference; indeed, their damage induction is in line with that by reference photon irradiations^12^. This allows a quick RBE calculation for DNA damage induction with the presented formulas. RBE for DSB classes therefore shows the same “overkill” effect as a function of LET. This can be also understood as follows: fewer densely ionizing particles are needed to deposit energy in the nucleus, corresponding to fewer damage sites. The internal complexity of these damages is however larger and keeps increasing with LET: this has been shown in^11^, where e.g. DSB multiplicity (defined as the number of DSBs per DSB cluster) was studied as a function of LET. When correlating these results to survival probability, one could conclude that high LET irradiation produces higher DNA damage than required for cell inactivation, hence the experimentally observed reduction in biological effectiveness. The “overkill” effect is therefore reproduced by PARTRAC MCTS calculations without the need for any ad hoc saturation correction. This is also the case for RBE calculation within the MSA approach^43^, where RBE is found to decrease for LET values higher than ~ 100 keV/μm. It has to be recalled that the PARTRAC MCTS calculations presented here reproduce the initial DNA damage only, which merely set the scene for DNA damage response and cell survival. Nevertheless, by looking at how different damage types depend on LET, and based on experimental RBE variation for cell survival, one may also try to identify the initial damage patterns leading to lethal events^11,44,45^.

## Conclusion

Analytical formulas have been proposed and their parameters adjusted to represent a comprehensive dataset of PARTRAC track-structure simulations on the yields of DNA strand breakage, single- and double-strand breaks, their clusters and sites upon irradiation with ions from hydrogen to neon at energies from 512 MeV/u down to stopping. This approach increases the potential of application of PARTRAC MCTS results. Analytical formulas provide a simple tool to be used by the research community working in this field, either as input parameters for modelling DNA damage repair or cell survival, or for comparison and benchmarking with other simulation codes under development. Furthermore, they provide a simple methodology by which macroscopic radiation transport codes can be extended to predict biological effectiveness. In this way, one can obtain from macroscopic codes also results related to subcellular levels, without the need of a software replica of the biological target and of running dedicated Monte Carlo simulations. This also implies sparing time and calculation resources. Due to the additivity of DNA damage (up to doses of the order of 100–1000 Gy), this strategy offers a quick way to obtain biological effectiveness in absolute terms as well as in terms of RBE for any mixed radiation field. Potential applications of such an approach include the mixed field generated by an ion beam in an oncological patient undergoing hadron therapy, thus supporting therapy planning optimization, or the one encountered by an astronaut in a deep-space mission, thus contributing to risk estimation for space radiation protection.
